# Adenomatoid Tumor of the Pericardium: A Case Report and Literature Review

**DOI:** 10.7759/cureus.81734

**Published:** 2025-04-04

**Authors:** Mokhtar H Abdelhammed, Nisha S Ramani

**Affiliations:** 1 Department of Pathology and Immunology, Baylor College of Medicine, Houston, USA

**Keywords:** adenomatoid tumour, benign lesion, mesothelial, pericardium, rare tumors

## Abstract

Adenomatoid tumors (ATs) are rare benign neoplasms of mesothelial origin, commonly found in the genital tracts but occasionally reported in extragenital locations, including the thoracic cavity. We describe the second case of a pericardial AT in a 65-year-old male with multiple comorbidities, including end-stage renal disease, diabetes mellitus, and heart failure. A small pericardial nodule was incidentally discovered during coronary artery bypass graft surgery without any clinical evidence of pericardial thickening. Histopathologic evaluation showed a well-circumscribed nodule with solid nests of epithelioid to spindled cells with bland cytology within a fibrous stroma. Immunohistochemical analysis demonstrated strong and diffuse positivity for calretinin, D2-40, WT-1, and pan-cytokeratin, confirming mesothelial differentiation. Markers of epithelial origin like MOC-31 and BerEp4 were negative, excluding the possibility of carcinomas of unknown origin. BAP1 nuclear expression was retained, and fluorescence in situ hybridization (FISH) for p16 (*CDKN2A*) was negative for homozygous deletion, distinguishing the lesion from mesothelioma. Besides contributing to the limited body of knowledge on extragenital and thoracic ATs, this case adds to the limited number of reports on pericardial ATs, providing an understanding of the rare occurrence of these tumors arising from the pericardium.

## Introduction

Adenomatoid tumors (AT) are benign neoplasms of mesothelial origin lining the walls of body cavities and covering internal organs. They typically occur in the genital tracts of males and females. These tumors are mostly found in the epididymis in males and the uterus or fallopian tubes in females. Usually, these tumors are small and seen incidentally in the resections for unrelated conditions. Adenomatoid tumors typically demonstrate a benign clinical course with no evidence of recurrence after resection and do not need further treatment [1-3].

The mesothelial differentiation of these tumors is recognized from its histological appearance and a classic mesothelial immunophenotype. Despite this, several neoplasms are included in the differential diagnosis of these tumors, like neuroendocrine neoplasms, metastatic adenocarcinoma, vascular neoplasms, and malignant mesothelioma. It can be especially difficult when they are encountered in rare extragenital sites [4-6].

Extragenital ATs have been reported in visceral organs and body cavity linings, including the liver, pancreas, adrenal glands, and peritoneum. Thoracic cavity involvement by this rare entity can occur and has only been described in nine patients so far. These cases are reported in the mediastinum and pleura, and there is a single case reported in the pericardium [4-11]. Here, we describe the second case of pericardial AT. 

## Case presentation

A 65-year-old male with a history of hypertension, end-stage renal disease, diabetes mellitus, and heart failure presented with exertional chest pain, dyspnea, lower extremity claudication, and dizziness. Cardiology evaluation revealed severe multivessel coronary artery disease, confirmed by left heart catheterization. He was referred for CABG x 3, with a free left internal mammary artery (LIMA) to the left anterior descending artery (LAD), saphenous vein graft (SVG) to obtuse marginal (OM), and saphenous vein graft (SVG) to the distal right coronary artery (RCA) with intraoperative transesophageal echocardiography (TEE) and epiaortic ultrasound (EAU). An incidental 1.3 cm pericardial nodule was found during the CABG procedure.

**Figure 1:**
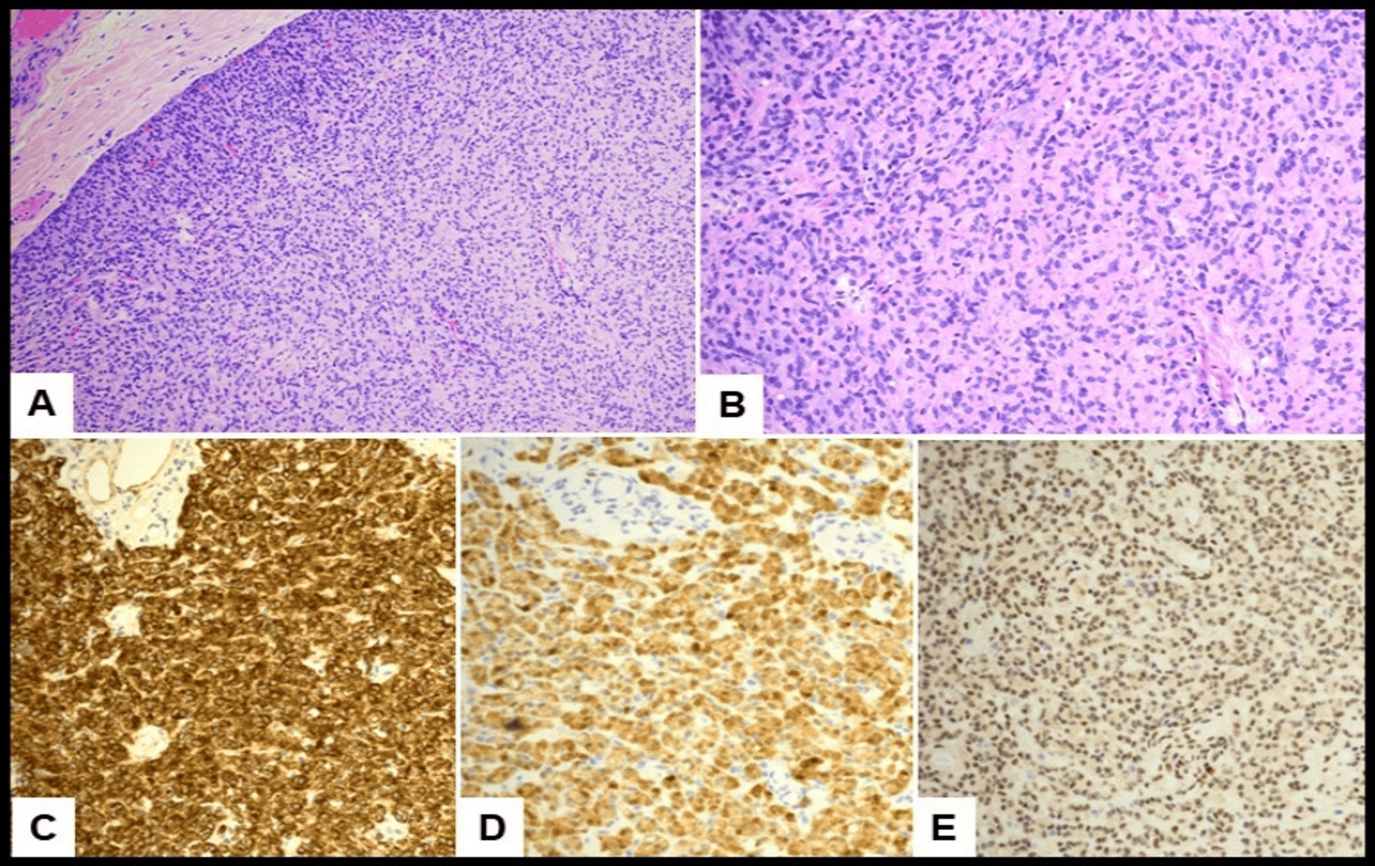
Histopathological and immunohistochemical examination of AT on hematoxylin and eosin (H&E). Well-circumscribed nodule with solid nests and cords of cells (A: H&E, 10X). The lesional cells appear bland with epithelioid-spindled morphology and abundant eosinophilic cytoplasm. There is no significant cytologic atypia or mitotic activity (B: H&E, 20X). The lesional cells show positivity for pancytokeratin (C: 20X) and calretinin (D: 20X), indicating mesothelial origin. Retained nuclear expression of BAP1 (E, 20X) is consistent with a benign mesothelial lesion

Gross examination revealed a small, oval, well-circumscribed tan-pink nodule without necrosis or cystic spaces. Microscopically, it showed a well-circumscribed lesion with a more solid appearance and epithelioid to spindled cells within a fibrous stroma (Figure 1A). Tumor cells were bland and low cuboidal with abundant eosinophilic cytoplasm and rare cystically dilated spaces (Figure 1B). No infarction, necrosis, calcification, cystic changes, atypia, or mitotic figures were noted. Immunohistochemical staining showed the lesional cells to be strongly positive for pan-cytokeratin (Figure 1C), calretinin (Figure 1D), WT-1, and D2-40, consistent with mesothelial differentiation. Immunostaining for BAP1 showed retained nuclear expression, consistent with a benign process (Figure 1E). 

The tumor cells were negative for synaptophysin, chromogranin, S100, MOC-31, TTF-1, p40, CDX2, and ERG, effectively excluding neuroendocrine, neural, or melanocytic differentiation; carcinomas from the lung or gastrointestinal primaries; and vascular lesions. Fluorescence in situ hybridization (FISH) for p16 (CDKN2A) was negative for homozygous deletion. The collective picture of morphology and immunophenotype was consistent with the diagnosis of adenomatoid tumor.

Subsequently, the patient’s health declined with worsening multiorgan failure, and he passed away after four months due to other comorbidities.

## Discussion

Despite the benign nature of ATs, they can sometimes mimic malignant conditions, leading to diagnostic challenges. ATs occur in both males and females, with no apparent gender predilection. The median age for patients with uterine or fallopian tube tumors is typically around 44 years and 56 years, respectively [1]. On the contrary, the majority of patients with thoracic ATs were females, highlighting a potential female predominance [4-11]. The ages cluster predominantly in the 50s and 60s, suggesting that thoracic ATs are more commonly observed in middle-aged to elderly individuals.

The tumor's typical localization includes the uterus, fallopian tubes, and ovaries [1]. Although predominantly found in the genital tract, they can also arise in other body cavities, though they are rarer. Thoracic AT is exceptionally uncommon, with only nine cases described in the literature. Reported cases of thoracic ATs are identified commonly in the mediastinum (n=4) and pleura (n=4), with a single case in the pericardium (n=1). Thoracic ATs ranged from 0.5 cm to 5.5 cm, with the pleural cases tending to be smaller (0.5-2.5 cm), while those in the anterior mediastinum were larger (4.3-5.5 cm) [2,6-8]. The previously reported pericardial case, the present case, and the posterior mediastinum case all displayed intermediate sizes (1.0-2.3 cm) [5,11].

Regardless of their site, ATs are usually discovered incidentally during imaging or surgeries for unrelated conditions and thus are often asymptomatic. Thoracic ATs are typically asymptomatic; however, chest pain was reported in a case involving a tumor larger than 5 cm, suggesting a potential correlation between tumor size and clinical presentation due to the limited space within the mediastinum with larger tumors [8].

A PubMed English literature review of thoracic AT, including the patient demographics, immunohistochemical markers, recurrence, and follow-up, is summarized in Table 1. There are eight studies reported so far on thoracic AT that include nine patients, ranging from 37 to 71 years, with a mean age of 59 years. The majority of these cases are reported in females (7/9, 77.7%). Mediastinum and pleura are the most reported sites (4/9, 44.4%), and our case marks the second case of pericardial AT. None of these cases showed recurrence in the follow-up period that ranged between 4 and 19 months.

**Table 1:** English literature review of eight studies on thoracic adenomatoid tumor (AT) Ref: Reference, F: Female, M: Male, N/A: Not available EMA: Epithelial membrane antigen, CEA: Carcinoembryonic antigen, TTF-1: Thyroid transcription factor-1, BER-EP4: Anti-human epithelial antigen, LEU-M1: Monoclonal antibody to Leu-M1, a granulocyte-related differentiation antigen, AE1/AE3: Mixture of 2 different clones of anticytokeratin monoclonal antibodies (AE1 and AE3), CAM 5.2: Cytokeratin CAM 5.2 low molecular weight keratin, MOC-31: Epithelial specific antigen, SP-A: Surfactant protein A, PAX8: Paired box gene 8, GLUT-1: Glucose transporter 1 (GLUT1)

Study [Ref]	Age (Years)/Sex	Site/Size	Immunohistochemical Markers	Recurrence	Follow-up
Kaplan et al. [2]	71/F (case 1)	Pleura/2.5 cm	Cytokeratin (+). CEA, LeuM1, BER-EP4 and B72.3 (-) CD34 and Factor VIII high-lighted vasculature (case 1)	None	8 months
	62/F (case 2)	Pleura/0.5 cm		None	12 months
Natarajan et al. [3]	53/M	Pericardium/1.0 cm	AE1/AE3 (+). CEA, BER-EP4, LEU-M1, CD34, factor VIII, periodic acid-schiff (PAS) and mucicarmine (-)	None	18 months
Umezu et al. [4]	70/M	Visceral pleura/0.4 cm	Calretinin, thrombomodulin, keratin, cytokeratin and vimentin (+). EMA, CEA, CD15, S-100, SP-A and p53 (-). Ki-67 7.6%.	N/A	8 months
Isotalo et al. [5]	66/F	Mediastinal Lymph Node/0.9 cm	Calretinin, CAM 5.2, AE1/AE3, CK7, and vimentin (+). Weak focal staining for CK5/6. CD15, CD31, CD34, CK20, MOC-31, and p-CEA (-)	N/A	N/A
Plaza et al. [6]	56/F	Anterior Mediastinum/5.5 cm	AE1/AE3, CK5/CK6, and calretinin (+). CK7, CK20, a-fetoprotein (AFP), CD31, CEA, MOC31, and chromogranin (-)	None	12 months
Minato et al. [7]	54/F	Pleura/0.8 cm	AE1/AE3, CAM5.2, vimentin, WT-1, calretinin, CK5/6, D2-40, and Thrombomodulin (+). CEA ,TTF-1, EMA, LeuM1, desmin, CD31, CD34, and GLUT-1 (-). p53 focal and weakly positive. Ki-67 1–2%	None	14 months
Goto et al. [8]	67/F	Anterior Mediastinum/ 4.3 cm	CAM5.2, calretinin, CK7, CK19, WT-1, HBME-1, and high molecular weight cytokeratin (+). Focal D2-40. CEA, a-fetoprotein (AFP), and p63 (-). Ki-67 1–2 %	None	19 months
Parekh et al. [9]	37/F	Posterior Mediastinum/ 2.3 cm	Pan-cytokeratin, calretinin, and WT1 (+). TTF1, CD31, CD34, PAX8, p53, and chromogranin (-). Ki-67 <2%	None	N/A

Immunohistochemical analysis across the nine patients consistently demonstrated strong positivity for AE1/AE3, pan-cytokeratin, and calretinin, with additional markers, including WT-1, CAM5.2, and CK5/6 showing positivity in select cases. Negative staining was noted for markers indicative of neuroendocrine (chromogranin, synaptophysin), vascular (CD31, CD34), and epithelial differentiation (CEA, MOC-31, CK20) in most patients. Ki-67 proliferation indices were reported in only two patients and were uniformly low (1-2%) with one outlier at 7.6%.

The etiology of AT remains largely unknown, although several factors have been implicated in their development. Some studies suggest that hormonal factors may play a role in their development. For instance, there has been a weak and focal expression of estrogen and progesterone receptors in some uterine adenomatoid tumors, hypothesizing a possible hormonal influence [1]. However, no definitive etiological factors have been identified. In terms of pathogenesis, ATs are genetically characterized by missense mutations in the TRAF7 gene, which drive aberrant NF-kB pathway activation [12].

Histopathologically, ATs show a mix of tubular, slit-like, or cystic spaces interspersed with solid areas. The tumor cells, which are typically cuboidal or flattened, exhibit minimal atypia. Occasionally, signet-ring cells, vacuolated lipoblast-like cells, and a lymphoid infiltrate can be seen [1-6]. Immunohistochemically, ATs express mesothelial markers, including calretinin, WT-1, CK5/6, and D2-40 along with cytokeratin AE1/AE3 and CAM5.2, which can aid in diagnosis. MOC-31, Ber-EP4, and CEA are useful epithelial markers to exclude the possibility of carcinomas of unknown origin. They show negative staining for markers associated with neuroendocrine (chromogranin, synaptophysin), vascular (CD31, CD34), and epithelial differentiation (CEA, MOC-31, CK20). Ki-67 generally shows low proliferative activity.

Although benign, ATs must be distinguished from mesotheliomas, which can present with similar features. Retention of nuclear BAP1 expression differentiates ATs from mesotheliomas, which often show BAP1 loss. Furthermore, TRAF7 mutations in ATs are associated with strong L1 cell adhesion molecule (L1CAM) expression, a marker of NF-kB pathway activation, which is absent in normal mesothelial cells and mesotheliomas [10]. This ImmunoProfile helps identify ATs with TRAF7 mutations from other mesothelial conditions. Additionally, unlike mesotheliomas, ATs lack CDKN2A and NF2 deletions, assisting in distinguishing them from other mesothelial entities [12]. Mesothelial hyperplasia is another differential, requiring histology and immunophenotype to distinguish it from ATs. Both involve bland mesothelial cell proliferation. However, ATs are characterized by well-circumscribed nodules of epithelioid cells forming tubulocystic spaces with vacuolated cytoplasm, as seen in this case. While both share positivity for mesothelial markers, features like lymphoid aggregates and a well-circumscribed margin, commonly seen in female genital tract ATs, aid in their differentiation.

## Conclusions

We present the second case of a pericardial AT in a 65-year-old male patient. The tumor was located in the pericardium and was discovered incidentally. Our case, along with the first reported instance of a pericardial AT, occurred in elderly male patients, suggesting a potential age-related predisposition. Furthermore, findings in thoracic ATs suggest a potential association between tumor size and location, with anterior mediastinal tumors showing a tendency to reach larger dimensions.

However, due to their rarity, a larger sample size is necessary to confirm these observations. Besides this case contributing to the limited knowledge on the rare entity of extragenital and thoracic ATs, it adds to the limited number of reports on pericardial ATs, providing an understanding of the rare occurrence of these tumors arising from the pericardium.
